# Study of the Regulatory Mechanism of miR-26a-5p in Allergic Asthma

**DOI:** 10.3390/cells12010038

**Published:** 2022-12-22

**Authors:** Jinnan Zhong, Min Liu, Shi Chen, Shuang Liu, Fajiu Li, Chenghong Li

**Affiliations:** Respiratory Critical Department, Wuhan No. 6 Hospital, Jianghan University Affiliated Hospital, Wuhan 430015, China

**Keywords:** allergic asthma, miR-26a-5p, fibrosis, target genes, airway remodeling

## Abstract

Objective: Allergic asthma is a growing burden on national public health services due to its high prevalence. The aim of this experiment was to investigate whether miR-26a-5p affects cellular fibrosis and thus airway remodeling in asthmatic mice through the regulation of target genes. Methods: Screening for differentially expressed miRNAs in asthma model mice was carried out by constructing a mouse model of allergic asthma. qRT-PCR was performed to determine candidate miRNAs in each group of bronchial tissues. Western blot detection of the expression levels of predicted candidate target genes in each group of bronchial tissues was conducted. A dual luciferase assay was performed to validate the binding of miR-26a-5p to target genes. Fibronectin, a marker of cellular fibrosis, was detected via flow cytometry. CCK8 and BrdU staining were used to detect the proliferation ability of each group of cells. Results: miR-26a-5p is able to target and bind to ABL2 3′-UTR, MMP16 3′-UTR and PDE7A 3′-UTR sequences. After interference with miR-26a-5p, improved bronchial histopathology and reduced peribronchial collagen deposition were found. Compared with the model group, interference with miR-26a-5p reduced lung fibrosis, decreased fibroblasts and increased apoptosis in mouse bronchial tissues; overexpression of miR-26a-5p decreased apoptosis in mouse bronchial tissues. Compared with the model group, the serum levels of IL-4, IL-5, IL-13 and I IFN-γ were decreased in the miR-26a-5p inhibitor group and increased in the miR-26a-5p mimic group. The immunohistochemical results showed that the expression of ABL2, MMP16 and PDE7A was significantly reduced after intervention with miR-26a-5p. Compared with the model group, the apoptosis rate of cells in the miR-26a-5p inhibitor group of the allergic asthma model was upregulated, the levels of IL-4, IL-5, IL-13, IFN-γ and ROS were decreased, the expression of the miRNA and proteins of ABL2, MMP16 and PDE7A was decreased, the expression of LC3A and P62 was significantly increased and the expression of LC3B, Beclin1, Atg5 and fibrosis markers collagen I and α-SMA was decreased. Conclusion: miR-26a-5p affects cellular fibrosis and thus airway remodeling in asthmatic mice by regulating target genes.

## 1. Introduction

As the most common chronic respiratory disease, asthma is characterized by wheezing and airflow obstruction [1]. Allergic asthma is a growing burden on national public health services due to its high prevalence. Airway remodeling is an important feature of asthma patients with chronic, persistent symptoms and occurs through a process that includes airway smooth muscle cell proliferation and subepithelial fibrosis [2,3]. Subepithelial fibrosis is mainly associated with the deposition of subepithelial collagen fibers, which are mainly secreted by fibroblasts [4]. Airway remodeling is a major cause of refractory asthma and irreversible damage to the asthma airway. Despite the large number of studies, the specific mechanisms regulating airway hyper-responsiveness and airway remodeling are unclear, and more research is needed at the molecular level to investigate the pathogenesis of asthma [5]. It has been found that ceramide can affect the severity of asthma by affecting cell apoptosis, the production of reactive oxygen species (ROS) and neutrophil infiltration after allergen attack [6]. Apoptotic epithelial cells have been reported in bronchial biopsies of adult patients with chronic, persistent asthma [7,8]. ROS are essential for initiating inflammatory responses, and allergic asthma is associated with increased ROS [9]. Autophagy can participate in the pathogenesis of asthma by regulating the body’s innate and adaptive immune responses [10].

MicroRNAs (miRNAs) are small, single-stranded nucleotide RNAs about 22 nt long that are found in a variety of organisms and play an important role in the regulation of gene expression [11]. Studies have shown that microRNAs are key regulatory RNAs in many diseases such as asthma and can be involved in pathophysiological processes such as airway remodeling by regulating multiple signaling pathways [12,13,14]. miRNAs play an important role in development, reproduction, apoptosis and pathogenesis by regulating the post-transcriptional expression of target genes [15]. miR-192-5p was found to reduce the inflammatory response in asthmatic mice, including lower levels of ovalbumin (OVA)-specific IgE, interleukins IL-4, IL-5 and IL-13, iNOS and COX-2, and its inhibitory effect on airway remodeling in asthmatic mice was achieved by reducing fibroblast growth factor-23 (FGF-23) levels, lowering MMP-2 and MMP-9 concentrations and downregulating type I collagen deposition [16]. In addition, miR-155 overexpression has been shown to be involved in the development of asthma and the activation of allergy-promoting cells [17]. Therefore, in-depth research on microRNAs can help to explore the pathogenesis of diseases and find new pathways for their early diagnosis and treatment.

In addition, a number of studies have found that microRNAs can be involved in the asthma process through target genes. miR-34/449 overexpression inhibits autophagy, reduces fibrosis, activates Akt and downregulates fibrosis-related factors, pro-inflammatory cytokines and nuclear factor-κB by targeting IGFBP-3 [18]. miR-133a plays a key role in asthma airway remodeling by targeting IGF1R through the PI3K/AKT/mTOR signaling pathway to regulate α-SMA expression [19]. Studying the mechanism of action of miRNAs and their target genes in asthma has important implications for the treatment of allergic airway diseases. In this study, we screened for differentially expressed miRNAs in asthma model mice, and bioinformatics analysis was conducted to screen for and validate the binding of target genes that interact with miRNAs related to airway inflammatory responses, airway remodeling and other pathological change processes in asthma. Animal asthma models and lung epithelial cell fibrosis models were constructed. By overexpressing miR-26a-5p, interfering with lentivirus and infecting animals and cells, we verified whether this miRNA affects cell fibrosis by regulating target genes, and thus affects airway remodeling in asthmatic mice.

## 2. Materials and Methods

### 2.1. Main Materials and Reagents

The following is a list of the main materials and reagents used in this study: OVA (A5253, sigma, Ronkonkoma, NY, USA); BCA Protein Concentration Assay Kit (PC0020, solarbio, Beijing, China); Oligo (dT)18/miR-RT Primer (3806, TAKARA, Kusatsu, Japan); PrimeScript II Rtase (2690A, TAKARA, Japan); T4 DNA ligase (Fermentas, Waltham, MA, USA); Dual luciferase reporter gene assay kit (RG027, Beyotime, Shanghai, China); Masson Trichrome Stain Kit (G1340, Solarbio, Beijing, China); ELISA kits IL-4 (HM10380), IL-5 (HM10213), IL-13 (HM10191) and IFN-γ (HM10058), purchased from Bioswamp(Wuhan, China); DAB Concentrate Kit (DA1010, Solarbio, Beijing, China); AnnexinV-PE/7 AAD Apoptosis Assay Kit (559763, BD, Becton Drive Franklin Lakes, NJ, USA).

### 2.2. Construction of an Allergic Asthma Model in Mice

A total of 30 female C57 strain mice, SPF class, 5–8 weeks old, were used in this study. All animals were sourced from Three Gorges University and housed in specific pathogen-free conditions. An amount of 200 μL of OVA (40 μg of OVA emulsified with 2 mg of aluminum hydroxide dissolved in 200 μL of sterilized PBS) was injected intraperitoneally on days 1 and 14. On days 28–31, 5% OVA was nebulized for 30 min daily, followed by an intranasal injection of 20 μL of OVA (40 mg/mL). The animals were then anesthetized using 1% pentobarbital, and bronchial tissue was taken.

### 2.3. qRT-PCR

qRT-PCR was used to detect candidate miRNAs in each group of bronchial tissues (the one with the greatest variability compared to normal mice was selected) and to predict the expression levels of candidate target genes. An amount of 100 mg of tissue sample was taken and homogenized in 1 mL of Trizol in a homogenizing tube. Total RNA was extracted and reverse-transcribed to synthesize cDNA, and PCR amplification was performed using cDNA as a template. The reaction procedure was as follows: 95 °C for 3 min; 95 °C for 5 s, 56 °C for 10 s and 72 °C for 25 s, for a total of 40 cycles. The PCR primers were synthesized by Wuhan Tianyi Huayu Gene Technology Co (Table 1). CT values were obtained at the end of the reaction, and statistical analysis was carried out using the 2^−∆∆CT^ method with GAPDH as an internal reference.

### 2.4. Screening for Target Genes

The miRNA target gene prediction tools miRDB, miRTarBase and Target Scan were used to calculate the predicted screened miRNA target genes. Target genes that appeared in all three database predictions were selected. The GO and KEGG pathways of the predicted target genes were further analyzed using the DAVID online tool, and genes associated with the pathological processes of asthma such as the airway inflammatory response and airway remodeling (predicted candidate genes) were selected for further analysis.

### 2.5. Western Blot

A Western blot assay was carried out to assess the expression levels of the predicted candidate target genes in each group of bronchial tissues. The tissue was cut into fine pieces, and lysate was added at a rate of 200 μL of lysate per 20 mg of tissue. The supernatant was taken after centrifugation, and the protein was quantified using the BCA protein concentration assay kit. Each well was sampled with 20 μg of protein and subjected to SDS-PAGE electrophoresis, and the proteins were separated and transferred to PVDF membranes and placed in 5% skimmed milk powder closed overnight at 4 °C. The primary antibody (ABL2, MMP16, PDE7A, caspase-9, caspase-3, Bax, Bcl-2 or GAPDH, 1:1000) was added and incubated for 1 h at room temperature. Then, the sample was washed 3 times with PBST, followed by the addition of HRP-labeled secondary antibody (Goat anti-Rabbit IgG, 1:20,000) and incubation for 1 h at room temperature. Afterwards, the sample was washed 3 times with PBST. ECL chemiluminescence reagent was added, the sample was placed in a fully automated chemiluminescence analyzer, and the protein bands were read in gray scale using TANON GIS software (Tanon, Shanghai, China).

### 2.6. Dual Luciferase Reporter Gene Test

Synthesis of the wt-ABL2 3′-UTR, MT-ABL2 3′-UTR, wt-MMP16 3′-UTR, MT-MMP16 3′-UTR, wt-PDE7A 3′-UTR and MT-PDE7A 3′-UTR genes was performed first. The vector was then digested with the target gene for 1–2 h at 37 °C. The vector was linked to the target gene. The DNA fragment to be transformed was added to a tube containing the top 10 receptor cells (50 μL of receptor cells requires 25 ng of DNA) in a volume that did not exceed 5% of the receptor cells. Finally, the plates were left to grow colonies for sequencing verification. Dual luciferase transfection was then performed to verify the binding of miR-26a-5p to ABL2, MMP16 and PDE7A. The experimental steps were as follows: Lysis of the cells was carried out. A sea kidney luciferase assay working solution was prepared by adding sea kidney luciferase assay substrate (100×) to sea kidney luciferase assay buffer at a ratio of 1:100. To assay the firefly luciferase activity F value (firefly luciferase), 100 µL of firefly luciferase assay reagent was added to each cell sample and mixed well, and the EP tubes were immediately placed in a fully functional microplate assay to calculate the F value. Then, 100 µL of sea kidney luciferase assay working solution was added, mixed well and immediately placed in a fully functional microplate assay, followed by the calculation of the sea kidney luciferase activity R value (*Renilla luciferase*). The relative luciferase activity was calculated from the detected F and R values.

### 2.7. Infection and Identification of miR-26a-5p Lentivirus

To explore the effects of miR-26a-5p interference and overexpression, pre-experiments were performed to map the MOI of the virus infection. Cells were inoculated 1 day prior to infection in 6-well plates at 3 × 10^5^ cells/well, 2 mL per well. Then, 1 mL of fresh medium was added prior to infection. After 8 h of incubation, the infected medium was replaced with 2 mL of fresh medium. After 72 h of cell infection, fluorescence expression was observed under a fluorescence microscope and photographed. Samples were collected for subsequent assays.

### 2.8. HE Staining

The mouse bronchial tissues were de-watered, waxed and embedded according to the usual procedure, as follows: The slices were 3 μm thick and stained with hematoxylin for 3 min. After washing with water, 1% hydrochloric acid in alcohol was applied for 1 s. After washing with water, the blue-promoting solution returned to blue for 5 s. After washing with water, the sample was stained with 0.5% eosin solution for 2 min. After washing with water, 80% ethanol was applied for 15 s, followed by 95% ethanol for 15 s, anhydrous ethanol for 2 s, xylene (I) for 2 s and xylene (II) for 2 s. Afterwards, neutral tree resin seal was applied. Finally, the sample was photographed using a microscope, and the Leica Application Suite image system was used to capture the relevant parts of the sample.

### 2.9. Masson Staining

The mouse bronchial tissues were de-watered, waxed and embedded according to the usual procedure, as follows: The slice thickness was 3 μm. Reagent (A) was stained for 5 min. Reagent (B) was partitioned and washed in water. Reagent (C) was returned to blue and washed with water. After washing with water, reagent (D) was stained for 1 min. Reagent (E) was washed for 1 min. Reagent (F) was washed for 2 min. Reagent (E) was washed for 1 min. Then, reagent (G) was directly stained for 1 min. Reagent (E) was washed for 1 min with 95% ethanol for rapid dehydration, followed by anhydrous ethanol (1) for 5 s, anhydrous ethanol (2) for 5 s, anhydrous ethanol (3) for 5 s, xylene (1) for 1 min, xylene (2) for 1 min and xylene (3) for 1 min. Neutral tree resin seal was applied. Finally, the sample was photographed using a microscope, and the Leica Application Suite image system was used to capture the relevant parts of the sample.

### 2.10. TUNEL Staining

The fixed mouse bronchial tissue was dehydrated and embedded in wax. The slice thickness was 3 μm. TUNEL staining was carried out after spreading and baking. The slides were baked in a constant-temperature oven at 65 °C for 1 h and then soaked in xylene I and xylene II. This was followed by incubation at 37 °C for 15 min with proteinase K working solution. Then, 50 μL of TUNEL reaction mix was added, followed by 50 μL of transformed POD and 50 μL of DAB substrate. The samples were then counterstained with hematoxylin. Finally, images of the samples were captured under a microscope.

### 2.11. ELISA

Dilutions of the standards were first performed. The enzyme label plate was set up with standard wells, blank wells and sample wells. An amount of 50 μL of standards at different concentrations was added to the standard wells, while 40 μL of sample followed by 10 μL of biotin-labeled antibody was added to the sample wells. Afterwards, 50 μL of enzyme reagent was added to each well except the blank wells, and then the plate was sealed with sealing film and incubated at 37 °C for 30 min. The concentrated washing solution was diluted 30 times with distilled water, carefully removing the sealing membrane and discarding the liquid from the wells. Then, 50 μL of color developer A and 50 μL of color developer B were added to each well, shaken gently, mixed well and developed for 10 min at 37 °C, protected from light. The reaction was terminated by adding 50 μL of termination solution to each well. The absorbance (OD) of each well was measured sequentially at 450 nm with a blank well set to zero.

### 2.12. Immunohistochemistry

The mouse bronchial tissues were de-watered, waxed and embedded according to the usual procedure, as follows: The thickness of the slices was 3 μm. The slides were then placed in a constant-temperature oven at 65 °C for 1 h, followed by soaking in xylene I for 15 min and then in xylene II for 15 min. After hydration, high-pressure (125 °C-103 KPa) repair was used for 18 min in 1 mM Tris-EDTA buffer solution. The slides were placed in 3% H_2_O_2_ and incubated for 10 min in a wet box. This was followed by incubation for 30 min in the sealed wet box. Primary antibodies ABL2 (1:400), MMP16 (1:200) and PDE7A (1:400) were added and incubated overnight at 4 °C. Secondary antibody MaxVision TM HRP-Polymer anti-Mouse/Rabbit IHC Kit was added and incubated for 60 min at 37 °C. This was followed by DAB staining. Hematoxylin re-staining was conducted for 3 min, followed by dehydration. The slides were placed in xylene for 3 min × 2 times and sealed with a neutral resin. The sample was photographed using a microscope, and the relevant parts of the sample were captured using the Leica Application Suite graphics system.

### 2.13. Construction and Characterization of a Cellular Allergic Asthma Model

The cells were divided into a control group and a model group. The cells were collected, the concentration of the cell suspension was adjusted with complete medium, and then the cells were divided into 6-well plates at 5 × 10^5^ cells/well, 2 mL per well, and incubated for 24 h at 37 °C in a 5% CO_2_ incubator. Cells were treated according to the grouping, and mouse lung TC-1 epithelial cells were treated with 20 ng/mL of IL-33 for 48 h. Cells were collected for subsequent assays.

### 2.14. CCK8

The experiment was divided into a control group, model group, model + miR-26a-5p-interfering lentivirus group, model + miR-26a-5p-overexpressing lentivirus group and negative control group. The cells were collected, the concentration of the cell suspension was adjusted, and then the cells were divided into 96-well plates at 3 × 10^3^ cells/well, 100 µL per well, and incubated overnight at 37 °C in a 5% CO_2_ incubator. The cells were treated according to the different groupings, and the culture was continued for 48 h. After removing the cell culture plate, 10 μL of CCK8 solution was added to each well and the culture was continued for 4 h. The absorbance value of each well was measured at 450 nm on an ELISA plate reader.

### 2.15. EdU Staining

EdU solution was diluted with complete medium at a ratio of 1000:1. An amount of 100 μL of 50 μm EdU medium was added to each well and incubated for 2 h. Then, 100 μL of cell fixative was added to each well and incubated for 30 min at room temperature. Following this, 2 mg/mL of glycine was added per well and incubated for 5 min in a decolorized shaker. Afterwards, 100 μL of PBS was added to each well and washed for 5 min in a decolorized shaker. Then, 100 μL of permeate (0.5% TritonX-100 in PBS) was added and incubated for 10 min in a decolorized shaker. This was followed by Edu staining and DNA staining. Finally, the sample was observed under a microscope.

### 2.16. Flow Cytometry

Each group of cells was collected, and then 1 × 10^6^ cells suspended in the culture medium were taken and centrifuged at 400× *g* for 5 min at 4 °C. Then, 1 mL of pre-cooled PBS was added, and the cells were mixed with gentle blowing and centrifuged at 400× *g* for 5 min at 4 °C. Following this, 10 μL of Annexin V-FITC and 10 μL of PI were added, mixed gently and incubated for 30 min at 4 °C, protected from light. Afterwards, 300 μL of PBS was added, followed by a flow-through assay. Analysis was carried out using NovoExpress analysis software.

### 2.17. Flow Antigen Testing

Each group of samples was taken, with 1 × 10^6^ cells, and 2 mL of PBS was added, followed by washing and centrifugation at 400× *g* for 5 min at 4 °C. Then, 1 mL of 4% paraformaldehyde was added for 30 min at room temperature, followed by washing, the addition of 200 μL of antibody diluted 1:500 with PBS and incubation for 1 h at room temperature. Following this, 200 μL of secondary antibody diluted 1:200 with PBS was added, followed by incubation at 37 °C for 1h. Cells were suspended in 400 μL of PBS, stored at 4 °C, protected from light, and assayed on the machine. The results of the experiments were analyzed using NovoCyte analysis software.

### 2.18. Statistical Data Analysis

One-way ANOVA and Duncan’s multiple comparisons were performed using SPSS 19.0 software (IBM, New York, NY, USA). The measurement data are expressed as the mean ± standard deviation. Comparisons between the two groups were performed using a *t*-test. A value of *p* < 0.05 was used to indicate statistically significant differences.

## 3. Results

### 3.1. Bioinformatic Analysis of mRNAs Interacting with miRNAs

**Figure 1:**
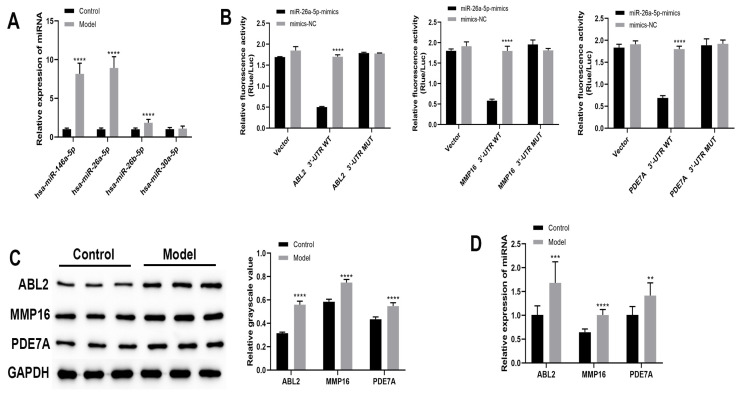
Bioinformatic analysis of mRNAs interacting with miRNAs. (**A**) qRT-PCR to detect expression of candidate miRNAs in each group of bronchial tissues. (**B**) Dual luciferase validation of miRNA binding to target genes. (**C**) Western blot to detect the expression of target genes. (**D**) qRT-PCR to detect the expression of target genes in each group of bronchial tissues. ** *p* < 0.01, *** *p* < 0.001, **** *p* < 0.0001.

We verified the expression of candidate miRNAs in each group of bronchial tissues using qRT-PCR (Figure 1A). The expression of miR-146a-5p, miR-26a-5p and miR-26b-5p was significantly higher in the model group compared with the control group (*p* < 0.0001). However, miR-26a-5p differed the most between the control and model groups. Therefore, we selected miR-26a-5p for follow-up experiments. The full results of the miRNA prediction are presented in Table S1. Then, we predicted the target gene of miR-26a-5p through raw letter analysis. The target genes that appeared in all three database predictions were selected (Table 2). The GO and KEGG pathways of the predicted target genes were further analyzed using the DAVID online tool, and genes related to the pathological change processes of asthma such as the airway inflammatory response and airway remodeling were selected (Table 3 and Table 4). Finally, we derived these miRNA target genes as ABL2, MMP16 and PDE7A. The binding site of miR-26b-5p to the target gene is shown in Figure S1. Then, we further verified the binding of miR-26a-5p to the target gene using a dual luciferase assay. The results revealed (Figure 1B) that the relative fluorescence activity ratio of TC-1 cells transfected with miR-26a-5p significantly elevated ABL2 3′-UTR, MMP16 3′-UTR and PDE7A 3′-UTR compared with mimic-NC (*p* < 0.0001). In addition, the expression of target genes in each group of bronchial tissues was detected through Western blotting and qRT-PCR. As shown in Figure 1C,D, the miRNA and protein expressions of ABL2, MMP16 and PDE7A were significantly higher in the model mice compared with the control group (*p* < 0.01). This result indicates that miR-26a-5p is able to target and bind to ABL2 3′-UTR, MMP16 3′-UTR and PDE7A 3′-UTR sequences.

### 3.2. Identification of Allergic Asthma Models in Mice

**Figure 2:**
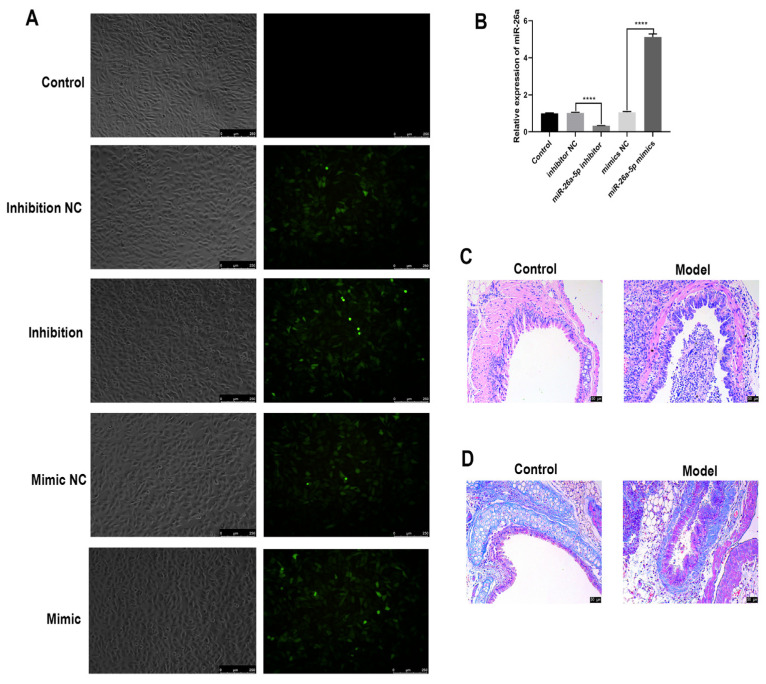
Transfection of miR-26a-5p lentivirus and identification of a mouse model of allergic asthma. (**A**) Fluorescence photography to observe the transfection of miR-26a-5p (scale bar: 250 µm). (**B**) PCR to detect the expression of miR-26a, **** *p* < 0.0001. (**C**) HE staining to observe the pathological changes in mouse bronchi (scale bar: 50 µm) (**D**) Masson staining to observe the fibrous changes in mouse bronchial tissues (scale bar: 50 µm).

As shown in Figure 2A, after the mimic, mimic-NC, inhibitor and inhibitor-NC, designed and synthesized from the miR-26a-5p sequence, were infected with TC-1 cells, a distinct green fluorescence was observed in the cells (Figure 2A). The expression of each group of miR-26a-5p was detected via PCR (Figure 2B). The expression of miR-26a-5p was significantly downregulated in the miR-26a-5p inhibitor group compared to the inhibitor-NC group (*p* < 0.0001). The expression of miR-26a-5p was significantly upregulated in the miR-26a-5p mimic group compared to the mimic-NC group (*p* < 0.0001). This indicates the successful transfection and normal expression of the marker gene. A mouse model of allergic asthma was then constructed. There was no significant collagen deposition around the bronchi in the control mice. Large peribronchial collagen deposits were seen in the model group, as shown in Figure 2C. Compared with the control group, the model group had severe lung fibrosis and increased fibroblasts (Figure 2D). This indicates that this allergic asthma mouse model was successfully constructed.

### 3.3. The Regulatory Role of miR-26a-5p in a Mouse Model of Allergic Asthma

**Figure 3:**
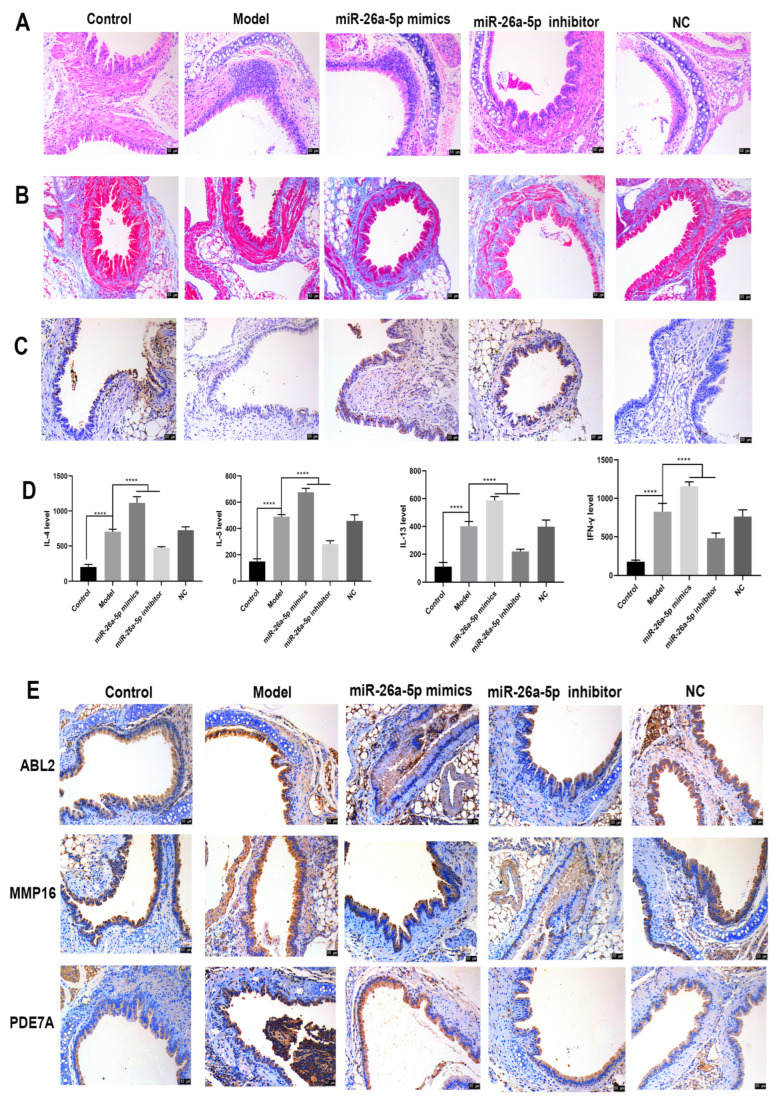
Regulatory role of miR-26a-5p in an allergic asthma mouse model. (**A**) HE staining to observe pathological changes in bronchial tissues of each group of mice (scale bar: 50 µm). (**B**) Masson staining to observe fibrous changes in bronchial tissues of mice (scale bar: 50 µm). (**C**) Apoptosis of bronchial tissue in each group detected via TUNEL staining (scale bar: 50 µm). (**D**) ELISA to detect IL-4, IL-5, IL-13 and IFN-γ levels in serum, **** *p* < 0.0001. (**E**) Immunohistochemistry to detect the expression of target genes in bronchial tissues (scale bar: 50 µm).

Histopathological changes in the bronchi of each group of mice were observed in HE staining (Figure 3A). In the control group, the bronchial epithelium was intact and structurally normal, and inflammatory cell infiltration was rare. The mice in the model group had a narrowed bronchial lumen, increased cupular cells in the mucosal epithelium, a thickened basal layer, hypertrophy of smooth muscle in the wall, storage of endobronchial secretions, increased microvascular permeability and increased collagen deposition around the bronchi. In contrast, the histopathological morphology of the bronchi was improved after interference with miR-26a-5p, and peribronchial collagen deposition was reduced. Next, Masson staining revealed severe pulmonary fibrosis and increased fibroblasts in the model group compared to the control group. Lung fibrosis and fibroblasts were reduced after interference with miR-26a-5p compared to the model group, as shown in Figure 3B. Additionally, the rate of apoptosis of bronchial tissue was higher in the model group compared to the control group. Compared with the model group, the rate of apoptosis in mouse bronchial tissues increased after interference with miR-26a-5p and decreased after overexpression of miR-26a-5p, as shown in Figure 3C. In addition, the serum levels of IL-4, IL-5, IL-13 and IFN-γ were measured via ELISA in mice (Figure 3D). The serum levels of IL-4, IL-5, IL-13 and IFN-γ were significantly higher in the model mice compared to the control group (*p* < 0.0001). Compared with the model group, the serum levels of IL-4, IL-5, IL-13 and IFN-γ were significantly lower in the miR-26a-5p inhibitor group (*p* < 0.0001), while the serum levels of IL-4, IL-5, IL-13 and IFN-γ were significantly higher in the miR-26a-5p mimic group (*p* < 0.0001). The expression of target genes in bronchial tissue was detected via immunohistochemistry (Figure 3E). The expression of ABL2, MMP16 and PDE7A was higher in the model group than in the control group, while the expression of ABL2, MMP16 and PDE7A was significantly reduced after intervention with miR-26a-5p. The results indicated that miR-26a-5p may affect pulmonary fibrosis, inflammation levels and apoptosis in allergic asthma mice by regulating target genes.

### 3.4. Construction and Characterization of a Cellular Allergic Asthma Model

**Figure 4:**
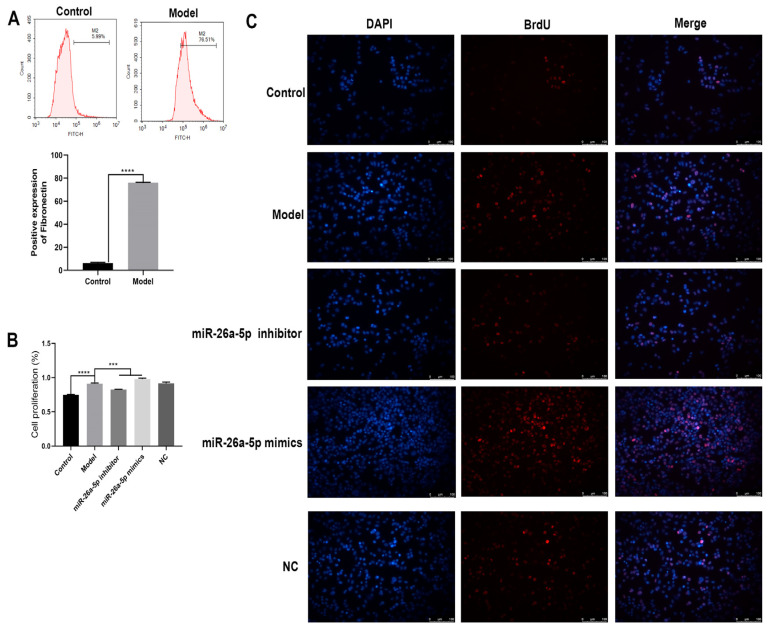
Cellular allergic asthma model construction and identification. (**A**) Flow detection of the cellular fibrosis marker fibronectin, **** *p* < 0.0001. (**B**) CCK8 detection of the proliferation ability of each group of cells, *** *p* < 0.001, **** *p* < 0.0001. (**C**) BrdU staining to observe the expression of each group (scale bar: 100 µm).

We constructed a cellular allergic asthma model using IL-33 induction. As shown in Figure 4A, the expression of the fibrosis marker fibronectin was significantly upregulated in the model group compared to the control group (*p* < 0.0001). Cell proliferation was examined for each group (Figure 4B). TC-1 cell proliferation was significantly increased in the model group compared to the control group (*p* < 0.0001). Compared with the model group, cell proliferation was significantly decreased in the miR-26a-5p inhibitor group (*p* < 0.0001) and increased in the miR-26a-5p mimic group (*p* < 0.0001). In addition, BrdU expression was higher in the model group than in the control group. Compared with the model group, the expression of BrdU was significantly reduced after interference with miR-26a-5p and increased after overexpression of miR-26a-5p. This suggests that miR-26a-5p could affect cell proliferation.

### 3.5. Effect of miR-26a-5p on Apoptosis and Inflammation in a Model of Allergic Asthma

**Figure 5:**
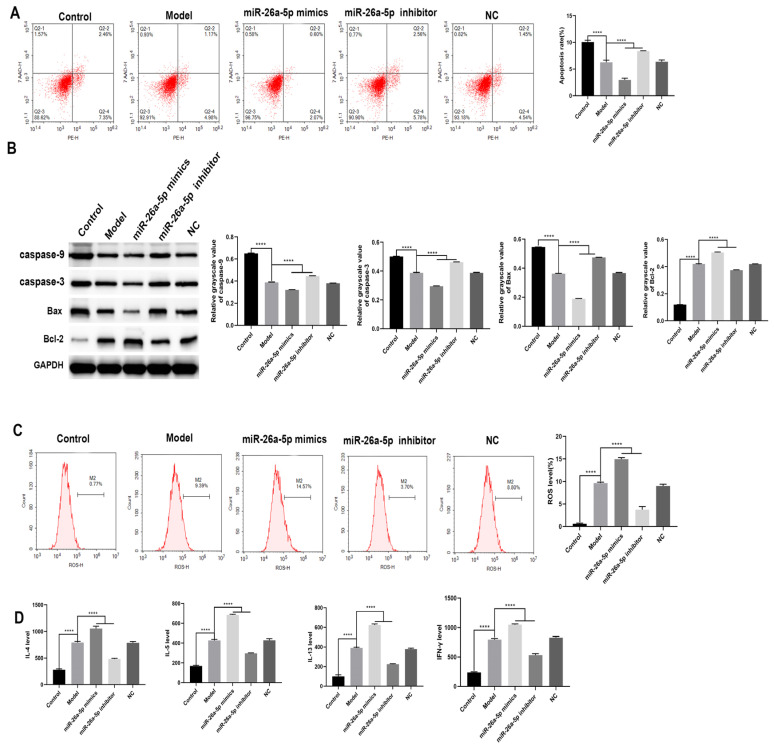
Effect of miR-26a-5p on apoptosis in an allergic asthma model. (**A**) Flow cytometry to detect the apoptosis rate in each group. (**B**) Western blot to detect the expression of apoptosis-related proteins caspase-9, caspase-3, Bax and Bcl-2 in each group. (**C**) Flow cytometry to detect the ROS level in each group. (**D**) ELISA detection of serum levels of IL-4, IL-5, IL-13 and IFN-γ. **** *p* < 0.0001.

We detected the apoptosis rate of each group of cells using flow cytometry (Figure 5A). The apoptosis rate of cells in the model group was significantly downregulated compared to the control group (*p* < 0.0001). Compared with the model group, the apoptosis rate of cells in the miR-26a-5p inhibitor group was significantly upregulated (*p* < 0.0001), and the apoptosis rate of cells in the miR-26a-5p mimic group was significantly downregulated (*p* < 0.0001). Concurrent assays for apoptosis-related proteins found consistent results demonstrating that interference with miR-26a-5p promoted apoptosis, as shown in Figure 5B. We then examined the ROS levels in each group of cells (Figure 5C). ROS levels were significantly higher in the model group cells compared to the control group (*p* < 0.0001). Compared with the model group, the ROS level of cells in the miR-26a-5p inhibitor group was significantly decreased (*p* < 0.0001), while the ROS level of cells in the miR-26a-5p mimic group was significantly increased (*p* < 0.0001). In addition, the levels of IL-4, IL-5, IL-13 and IFN-γ in the cell supernatant were measured via ELISA in each group of cells (Figure 5D). The levels of IL-4, IL-5, IL-13 and IFN-γ in the cell supernatant were significantly higher in the model group compared to the control group (*p* < 0.0001). Compared with the model group, the levels of IL-4, IL-5, IL-13 and IFN-γ in the cell supernatant in the miR-26a-5p inhibitor group were significantly lower (*p* < 0.0001), and the levels of IL-4, IL-5, IL-13 and IFN-γ in the cell supernatant in the miR-26a-5p mimic group were significantly higher (*p* < 0.0001). This suggests that miR-26a-5p can affect apoptosis, inflammation and ROS levels.

### 3.6. Effect of miR-26a-5p on Cellular Autophagy in a Model of Allergic Asthma

**Figure 6:**
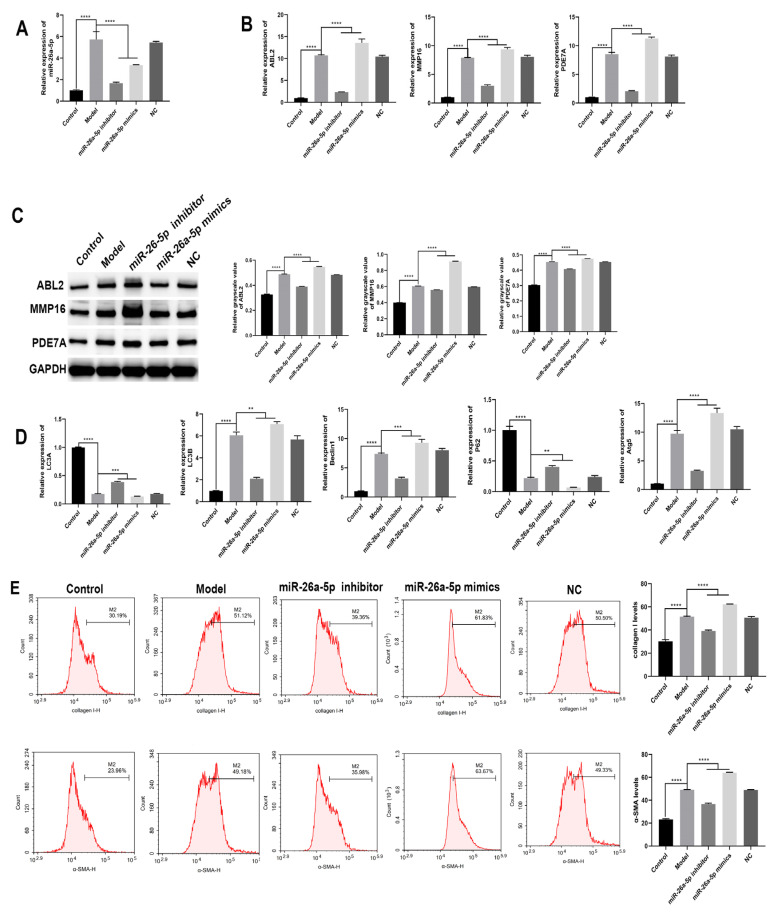
Effect of miR-26a-5p on autophagy in cells of allergic asthma model. (**A**) PCR to detect the expression of miR-26a-5P. (**B**) qRT-PCR to detect the expression of ABL2, MMP16 and PDE7A in each group of cells. (**C**) Western blot to detect the expression of target genes in each group of cells. (**D**) qRT-PCR to detect the expression of autophagy-related LC3A, LC3B, Beclin1, P62 and Atg5. (**E**) Detection of the levels of collagen I and α-SMA, the markers of cellular fibrosis. ** *p* < 0.01, *** *p* < 0.001, **** *p* < 0.0001.

As shown in Figure 6A, the expression of miR-26a-5p was significantly higher in the model group cells compared to the control group (*p* < 0.0001). The expression of target genes in each group of cells was detected via qRT-PCR and Western blotting (Figure 6B,C). The miRNA and protein expressions of ABL2, MMP16 and PDE7A were significantly higher in the model group compared to the control group (*p* < 0.0001). Compared with the model group, the miRNA and protein expressions of ABL2, MMP16 and PDE7A were significantly lower in the miR-26a-5p inhibitor group (*p* < 0.0001), while the miRNA and protein expressions of ABL2, MMP16 and PDE7A were significantly higher in the miR-26a-5p mimic group (*p* < 0.0001). In addition, we examined the expression of autophagy-related proteins (Figure 6D). Compared with the control group, the expression of LC3A and P62 was significantly decreased (*p* < 0.0001), while the expression of LC3B, Beclin1 and Atg5 was significantly increased (*p* < 0.0001) in the model group cells. Compared with the model group, in the miR-26a-5p inhibitor group, the expression of LC3A and P62 was significantly increased (*p* < 0.0001), while the expression of LC3B, Beclin1 and Atg5 was significantly decreased (*p* < 0.0001); in the miR-26a-5p mimic group, the expression of LC3A and P62 was significantly decreased (*p* < 0.0001), while the expression of LC3B, Beclin1 and Atg5 was significantly increased (*p* < 0.0001). In addition, each group of cellular fibrosis markers was detected via flow cytometry (Figure 6E). The expression of collagen I and α-SMA was significantly higher in the cells of the model group compared to the control group (*p* < 0.0001). Compared with the model group, the expression of collagen I and α-SMA was significantly lower in the miR-26a-5p inhibitor group (*p* < 0.0001), while it was significantly higher in the miR-26a-5p mimic group (*p* < 0.0001). This suggests that miR-26a-5p can affect cell fibrosis and autophagy by regulating target genes.

## 4. Discussion

Allergic asthma is usually defined as asthma associated with an allergy to an airborne allergen. Allergic asthma is the most common phenotype of asthma [20]. Allergic asthma is a Th2-driven process [21]. It mainly involves IL-4, IL-5 and IL-13 cytokines. IL-4 and IL-13 are essential for IgE class conversion [22]. In addition, changes in microRNA (miRNA) expression can contribute to the pathogenesis of many diseases, including asthma. miR-125b-5p, miR-223-3p and miR-26a-5p were identified as potential regulators that may contribute to the pathogenesis of asthma [23]. It has also been found that miR-155 can regulate the Th2 immune response and gene signaling [13]. Deletion of miR-155 leads to a decrease in activated CD4+ T cells, resulting in reduced production of Th2 cytokines (IL-4, IL-5 and IL-13) in the airways [24]. It was shown that miR-155 was significantly upregulated in an OVA-induced asthma mouse model, and that airway inflammation, airway hyper-responsiveness (AHR) and Th2 cytokine release were alleviated after lentiviral injection using miR-155 interference [17].

In this study, we first screened differentially expressed miRNA in asthma model mice as miR-26a-5p. Using bioinformatics analysis, the target genes interacting with miRNAs related to the airway inflammatory response, airway remodeling and other pathological change processes in asthma were screened as ABL2, MMP16 and PDE7A. A number of studies have found that microRNAs can be involved in the asthma process through target genes. miR-34/449 overexpression inhibits autophagy, reduces fibrosis, activates Akt and downregulates fibrosis-related factors, pro-inflammatory cytokines and nuclear factor-κB by targeting IGFBP-3 [18]. miR-133a plays an important role in asthma airway remodeling by targeting IGF1R and regulating α-SMA expression [19]. In this study, we found that intervention with miR-26a-5p expression improved the bronchial tissues of allergic asthmatic mice, reduced fibrosis and decreased the serum levels of IL-4, IL-5, IL-13 and IFN-γ. Meanwhile, miR-26a-5p was found to target and bind to ABL2 3′-UTR, MMP16 3′-UTR and PDE7A 3′-UTR sequences via a luciferase assay. The expression of ABL2, MMP16 and PDE7A was higher in allergic asthmatic mice than in normal mice. In contrast, the expression of ABL2, MMP16 and PDE7A was significantly reduced after intervention with miR-26a-5p expression. This demonstrates that miR-26a-5p may influence cellular fibrosis through the regulation of target genes, which in turn affects airway remodeling in asthmatic mice. We then constructed an allergic asthma cell model to further validate the effects of miR-26a-5p on apoptosis, ROS and inflammation levels in allergic asthma cells. Research has been able to demonstrate that apoptosis is a way to eliminate harmful cells and reduce inflammation, with the development of drugs that target eosinophil apoptosis being a possible strategy for the treatment of allergic asthma [25]. IL-5 plays a key role in eosinophil proliferation, differentiation, maturation, migration and apoptosis [26,27]. During allergic inflammation, eosinophils release reactive oxygen species (ROS) that cause tissue damage [28]. One study found that a drug (thyme) was able to relieve bronchial asthma by reducing ROS. This is consistent with the results of the present study demonstrating that interference with miR-26a-5p expression has an antioxidant effect on allergic asthma.

There are few studies on autophagy in allergic asthma. Increased LC3B expression in airway epithelial cells and elevated levels of Atg5 in lung homogenates were found in cockroach-allergen-induced allergic airway inflammation [29]. It has been hypothesized that dysregulation of basic cellular processes that maintain homeostasis and the physiological balance in the body may be a key clinical sign that leads to asthma [30]. Autophagy has been shown to play an important role in downstream changes triggered by allergens and respiratory infections [31,32]. Recent studies have linked autophagy to major signs of, for example, airway smooth muscle [33,34,35], the extracellular matrix (ECM) [36,37] and fibrosis [38]. Atg5 deficiency results in reduced pro-fibrotic signaling and ECM protein release [4]. Inhibition of miR-26a-5p expression in this study resulted in a significant increase in the expression of LC3A and P62, and a significant decrease in the expression of LC3B, Beclin1 and Atg5, in allergic asthma cells.

## 5. Conclusions

In this experiment, by constructing an allergic asthma mouse model and a cellular allergic asthma model, the most differential miRNA was screened as miR-26a-5p. It was also found that inhibition of miR-26a-5p expression could significantly improve the pathological state of bronchial tissue, reduce the degree of lung fibrosis, promote the apoptosis of bronchial tissue and reduce the level of inflammation. Meanwhile, inhibition of miR-26a-5p was able to affect the occurrence of autophagy in allergic asthma cells. It was also demonstrated that miR-26a-5p could target and bind to ABL2, MMP16 and PDE7A to affect cellular fibrosis and thus airway remodeling in asthmatic mice.

## Figures and Tables

**Table 1:** Primer sequences.

Primer Name	Sequence	Amplified Fragment Size (bp)
ABL2-F	CCGCCGTCGTGTTACTTT	119
ABL2-R	ATGGTTCTCGCCCACTAGG	
MMP16-F	CGTGCCTTTGATGTGTGG	184
MMP16-R	CGGGTCCAGGGAAATAAG	
PDE7A-F	AGTCTCCGCCAGGAACAT	125
PDE7A-R	CTTGTCCATTGTAATCCTCATC	
miR-146a-5p-F	CTGGTGTCGTGGAGTCGG	57
miR-146a-5p-R	GGGGTGAGAACTGAATTCCA	
miR-26a-5p-F	CTGGTGTCGTGGAGTCGG	57
miR-26a-5p-R	GGGGTTCAAGTAATCCAGGA	
miR-26b-5p-F	CTGGTGTCGTGGAGTCGG	57
miR-26b-5p-R	GGGGGTTCAAGTAATTCAGG	
miR-30a-5p-F	CTGGTGTCGTGGAGTCGG	57
miR-30a-5p-R	GGGGTGTAAACATCCTCGAC	
U6-F	CTCGCTTCGGCAGCACATATACT	93
U6-R	ACGCTTCACGAATTTGCGTGTC	
LC3A-F	CTGTCCTGGATAAGACCAAGTTT	171
LC3A-R	CCTGTTCATAGATGTCAGCGAT	
LC3B-F	ACCAAGCCTTCTTCCTCC	94
LC3B-R	CCGTCTTCATCTCTCTCACTC	
Beclin1-F	GAGAGACCCAGGAGGAAGA	116
Beclin1-R	GGGACTGAGGAATAGTAAGCA	
P62-F	TTGCCTTTTCCAGTGATGA	196
P62-R	TAGCGAGTTCCCACCACA	
Atg5-F	GGCTCACTTTATGTCGTGTATG	93
Atg5-R	AGCTGCTTGTGGTCTTTTTT	
GAPDH-F	CCTTCCGTGTTCCTAC	152
GAPDH-R	GACAACCTGGTCCTCA	

**Table 2:** Screening of target genes.

Gene Symbol	Gene Description
ABL2	c-abl oncogene 2, non-receptor tyrosine kinase
MMP16	matrix metallopeptidase 16 (membrane-inserted)
PDE7A	phosphodiesterase 7A

**Table 3:** Target Gene Association GO.

Gene Symbol	GO Description		
	Bp	Cc	Mf
ABL2	signal transduction	actin cytoskeleton	phosphotyrosine residue binding
MMP16	extracellular matrix disassembly	integral component of plasma membrane	metalloaminopeptidase activity
PDE7A	cAMP-mediated signaling	cytosol	3′,5′-cyclic-nucleotide phosphodiesterase activity

**Table 4:** Target gene associated with KEGG.

Gene Symbol	Pathway Name
ABL2	Ras signaling pathway
MMP16	Activation of matrix metalloproteinases
PDE7A	GPCR downstream signaling

## Data Availability

The data used to support the findings of this study are included within the article.

## Supplementary Materials

The following supporting information can be downloaded at: https://www.mdpi.com/article/10.3390/cells12010038/s1, Figure S1: Conserved; Table S1: miRNA.
