# Supplementary material for: Study of the Regulatory Mechanism of miR-26a-5p in Allergic Asthma
Source: Cells. 2022 Dec 22;12(1):38. doi: 10.3390/cells12010038 (PMC9818720; doi:10.3390/cells12010038)
Supplement: Supplementary file 1 [file cells-12-00038-s001.zip › English-Editing-Certificate-55805.pdf]

We certify that the following article

Study of the regulatory mechanism of miR-26a-5p in allergic asthma

Chenghong Li

has undergone English language editing by MDPI. The text has been checked for correct use of grammar and common technical terms, and edited to a level suitable for reporting research in a scholarly journal.
MDPI uses experienced, native English speaking editors. Full details of the editing service can be found at
► https://www.mdpi.com/authors/english.

MDPI Multidisciplinary Digital Publishing Institute

Basel, Switzerland
December 2022

AUTHORservices
