# Supplementary figures and images for: Study of the Regulatory Mechanism of miR-26a-5p in Allergic Asthma
Source: Cells. 2022 Dec 22;12(1):38. doi: 10.3390/cells12010038 (PMC9818720; doi:10.3390/cells12010038)

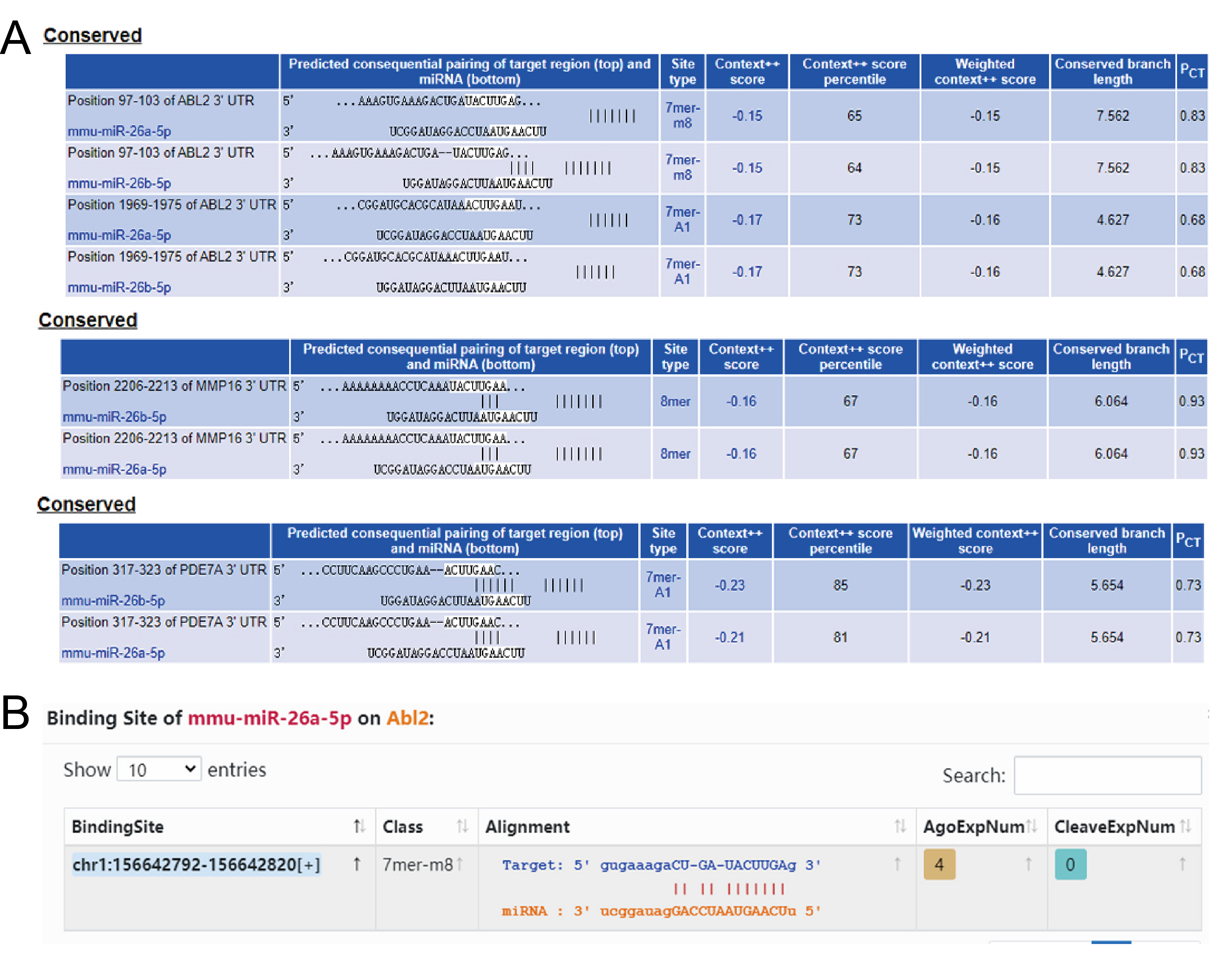

Supplement: Supplementary file 1 [file cells-12-00038-s001.zip › Fig.S1.jpg]
